# Hydrogen sulfide modulates gene networks in hypoxia/reoxygenation-stressed trophoblasts: insights from transcriptome profiling

**DOI:** 10.3389/fbinf.2026.1785302

**Published:** 2026-06-16

**Authors:** Shivani, Amit Katiyar, Sunil Kumar Gupta, Pallavi Arora, Kiran Kumari, Tooba Tahreem Khan, Pallavi Kshetrapal, Renu Dhingra

**Affiliations:** 1 Department of Anatomy, All India Institute of Medical Sciences, New Delhi, India; 2 Bioinformatics Facility, Centralized Core Research Facility, All India Institute of Medical Sciences, New Delhi, India; 3 Department of Anatomy, ASMC Kushinagar, Uttar Pradesh, India; 4 Department of Anatomy, Shri Mata Vaishno Devi Institute of Medical Excellence, Kakryal, India; 5 Department of Biotechnology, All India Institute of Medical Sciences, New Delhi, India; 6 Lab of Perinatal Research, BRIC-Translational Health Science and Technology Institute, Faridabad, Haryana, India

**Keywords:** cystathionine gamma lyase, hydrogen sulphide, hypoxia/reoxygenation injury, preeclampsia, trophoblast, transcriptomic

## Abstract

**Introduction:**

Hydrogen sulfide is an endogenous gaseous signalling molecule with recognized roles in vascular regulation, redox homeostasis, and inflammation. In the placenta, H_2_S is essential for maintaining trophoblast function and promoting healthy vascular remodelling. Impaired H_2_S signalling has been implicated in placental disorders characterized by oxidative stress, particularly in preeclampsia. One of the principal drivers of oxidative stress in the placenta is H/R injury, which mimics the intermittent perfusion patterns seen in early placental maldevelopment. Although the protective roles of H_2_S have been described in several ischemia-reperfusion models, its genome-wide transcriptional effects on trophoblasts under hypoxia/reoxygenation-induced oxidative stress remain unknown.

**Methods:**

HTR-8/SVneo trophoblasts were subjected to H/R injury induced by varying oxygen concentrations to model the fluctuating oxygen environments of early placental development, followed by treatment with an exogenous H_2_S donor (NaHS). A CSE inhibitor (PAG) treatment was also given. RNA sequencing was performed to characterize global gene expression changes. Differentially expressed genes were analyzed using KEGG and Gene Ontology enrichment, protein–protein interaction network mapping, and transcription factor prediction.

**Results:**

H/R induced extensive transcriptional remodelling, with robust activation of HIF-1, PI3K–Akt, MAPK, Rap1/Ras, NF-κB, and focal adhesion pathways. H/R [2/10% O_2_] triggered pronounced glycolytic, hypoxia-adaptive, anti-apoptotic, and pro-invasive signatures. NaHS modulated these responses in a context-dependent manner: it attenuated early chemokine-driven inflammation, enhanced angiogenic and ECM-remodelling programs, and strengthened metabolic adaptation under a higher hypoxic burden 2/10% H/R paradigm. PAG induced a chronic inflammatory angiogenic signature, indicating endogenous H_2_S restrains basal inflammatory activation. Integrated regulation of proliferation, migration, apoptosis, morphogenesis, and angiogenesis was observed through biological process analysis, with major changes noticed in NaHS-treated 2/10% H/R conditions. JUN, PTGS2, MAP3K5, DUSP1, SFN, NCF2, THBS2, and GADD45A emerged as the central interconnected hub-gene module through PPI analysis. Among these, JUN and PTGS2 appeared as potential integrators of trophoblast remodelling, redox stress, and inflammatory signalling.

**Discussion:**

Our study provides the first evidence of transcriptomic analysis showing that H_2_S alters gene networks in trophoblast cells subjected to H/R-induced oxidative stress. The results highlight coordinated regulation of metabolic, angiogenic, and inflammatory pathways, providing fundamental understanding into how H_2_S may influence trophoblast adaptation to stress.

## 1 Introduction

Hydrogen sulfide (H_2_S), an endogenous gaseous signalling molecule, is synthesized mainly by cystathionine γ-lyase (CSE) and related enzymes. It is crucial for vascular regulation, mitochondrial function, redox homeostasis, and modulating inflammatory signalling (Bir and Kevil, 2013). In the placenta, H_2_S facilitates trophoblast migration, invasion, and spiral artery remodelling, processes essential for favouring low-resistance uteroplacental circulation. Previous studies have shown that placentae from preeclamptic pregnancies display lower H_2_S bioavailability and reduced CSE expression, indicating impairment of this pathway in disease conditions (Wang et al., 2013).

Trophoblasts are extremely metabolically active cells that depend on precisely regulated availability of oxygen. Disrupted placental perfusion during the early gestational period leads to wavering oxygen tension, causing episodes of hypoxia followed by reoxygenation. Such hypoxia/reoxygenation (H/R) injury is mainly acknowledged as a central force behind placental endothelial activation, inflammation, oxidative stress, and apoptosis, all of which lead to the pathophysiology of preeclampsia (PE) (Hung et al., 2004). Using HTR-8/SVneo cells as an *in vitro* H/R model, these conditions have been mimicked, and elevated release of pro-inflammatory cytokines, disrupted trophoblast activity, increased sFlt-1, and decreased PlGF were observed, thereby creating a PE-like phenotype (Fuenzalida et al., 2022).

Despite the protective role of H_2_S in several models of ischemia/reperfusion injury and oxidative stress, including pro-migratory, amplification of mitochondrial activity, and as an anti-inflammatory agent, (Gupta et al., 2023; Liu et al., 2022), its distinct transcriptional influence on trophoblasts subjected to H/R-induced oxidative stress remains largely uncharacterized. The available literature has concentrated on discrete pathways or isolated molecular markers, which has left a research gap in how the global gene networks are regulated by H_2_S during placental H/R injury.

During the early trimester, the placenta undergoes a hypoxic environment that changes with arterial remodelling, which may induce various adaptive and maladaptive transcriptional responses. How H_2_S modulates these responses across a spectrum of hypoxic stress is vital for identifying its role in supporting placental stability.

Despite these advances, transcriptome-wide studies examining the influence of H_2_S on trophoblast gene expression under H/R injury are still lacking. Addressing this gap will enable identification of coordinated molecular programs that support trophoblast survival, promote angiogenesis, facilitate metabolic adaptation, and modulate inflammatory responses.

The present study utilizes high-throughput RNA sequencing to characterize global gene expression changes in HTR-8/SVneo trophoblasts exposed to H/R stress with or without H_2_S supplementation or CSE inhibition. By profiling pathway enrichment, protein–protein interaction networks, and transcriptional regulators, this work aims to provide the first comprehensive view of gene networks influenced by H_2_S in trophoblasts under oxidative stress, thereby generating mechanistic hypotheses for future functional and translational research.

## 2 Materials and methods

### 2.1 Cell culture

HTR-8/SVneo, first-trimester human trophoblast cells (ATCC, Manassas, VA, USA) were cultured in a 1:1 mixture of Dulbecco’s Modified Eagle Medium (DMEM; HyClone™ SH30243.01) and Ham’s F-12 medium (Sigma-Aldrich, N6658), supplemented with 10% fetal bovine serum (FBS; Gibco™ 10270160), 100 U/mL penicillin, 100 μg/mL streptomycin (Amresco, K952), and 250 μg/mL amphotericin B (Gibco™, 15290–018). Cultures were maintained at 37 °C in a humidified atmosphere containing 5% CO_2_.

### 2.2 Treatment groups and experimental approach

Cells were divided into the following experimental groups: (b) H/R [5/20%O_2_], (c) H/R [5/20%O_2_] with NaHS (100 µM), (d) H/R [2/10%O_2_], (e) H/R [2/10%O_2_] with NaHS (100 µM), (f) NaHS alone under normoxia (100 µM), and (g) DL-propargylglycine (PAG), a CSE inhibitor, under normoxia (100 µM for 24 h). Group (a) consisted of untreated normoxic controls. For treatment groups, NaHS was supplemented immediately following completion of the H/R cycles and incubated for 5 min.

H/R conditions were modelled using two oxygen paradigms: 2%→10% and 5%→20% O_2_. Exposure to 2% O_2_ recapitulates a low-oxygen environment characteristic of early placentation, which is critical for embryogenesis by maintaining reduced oxidative stress and regulating key trophoblast functions, including proliferation, differentiation, and invasion, while gradual reoxygenation at 10% O_2_ approximates oxygen levels in the intervillous space during later gestation (Burton et al., 2021). In contrast, 20% O_2_ corresponds to atmospheric oxygen levels routinely used in standard cell culture systems, although higher than physiological placental oxygen tension. Trophoblast cell lines are maintained under these conditions *in vitro*; thus, a transition from lower oxygen levels (2%–5%) to 20% O_2_ may represent a relative hyperoxic shift. This shift is widely used to model oxidative stress associated with H/R injury, mimicking key aspects of placental ischemia–reperfusion (I/R) implicated in PE, while 5% O_2_ represents moderate physiological oxygen tension and hypoxia with respect to 20%. The first H/R condition in our experimental protocol (group b) involved incubation at 5% O_2_ for 4 h followed by 20% O_2_ for 1 h, while the second H/R condition (group d) used 2% O_2_ for 4 h followed by 10% O_2_ for 1 h. Each H/R cycle was repeated twice to simulate recurrent I/R episodes. It is important to note that, unlike the gradual *in vivo* increase in placental oxygen tension (approximately 2%–8%) across early to mid-gestation, the abrupt oxygen fluctuations applied *in vitro* are designed to mimic pathological I/R like conditions, as observed in disorders such as PE.

NaHS was used as a hydrogen sulfide donor at concentrations of 25, 50, 100, and 150 µM with exposure durations of 5 and 10 min to determine optimal conditions. Based on preliminary experiments assessing cellular morphology and wound healing capacity, 100 µM NaHS for 5 min was selected for subsequent studies as it produced the most consistent and robust biological response (Gupta et al., 2023). This short exposure duration was chosen to capture early H_2_S-mediated signaling events, as H_2_S is a rapidly acting gaseous signaling molecule capable of inducing intracellular signaling responses within minutes, including activation of redox-sensitive pathways and kinase signaling cascades (Kimura et al., 2012; Sulen et al., 2016). The selection of 100 µM NaHS was guided by both experimental optimization and existing literature, where similar concentrations have been widely used to investigate H_2_S-mediated signalling (Zhang et al., 2012). All treatments were given in 1% FBS medium and performed in three independent biological replicates.

### 2.3 RNA extraction and quality control

Following treatment, total RNA was extracted using the Qiagen RNeasy Mini Kit (Cat No. 74106). Lysates were incubated at room temperature for 5 min, mixed with 0.5 volumes of absolute ethanol, and loaded into RNeasy spin columns. After centrifugation (8,000 rpm, 1 min) and flow-through removal, on-column DNase I digestion (Cat No. 79254) was performed. Columns were washed, and RNA was eluted with nuclease-free water.

RNA purity and concentration were measured using a NanoDrop spectrophotometer (Thermo Scientific 2000), integrity was assessed on the Agilent TapeStation, and concentration was confirmed with a Qubit RNA HS assay kit (Q32855).

### 2.4 Library preparation and RNA sequencing

RNA-seq libraries were prepared by Genotypic Technology Pvt. Ltd. (Bangalore, India) using the NEBNext® Ultra™ II Directional RNA Library Prep Kit (New England BioLabs, MA, USA). Each library was generated from 500 ng of total RNA, following poly(A) mRNA enrichment and strand-specific cDNA synthesis. Double-stranded cDNA was purified using JetSeq Beads (Bioline, BIO-68031), followed by end-repair, A-tailing, adapter ligation, and indexing PCR. Final libraries were quantified, size-profiled (Agilent 2200 TapeStation), and sequenced on the Illumina HiSeq X Ten platform (paired-end, 150 cycles).

### 2.5 Quality control and data processing

Raw reads were assessed with FastQC, and adapters/low-quality sequences were removed using Fastp (Chen et al., 2018). Clean reads were aligned to the human reference genome using HISAT2 (Kim et al., 2019). Gene-level read counts were obtained using HTSeq-Count (Anders et al., 2014). Differentially expressed genes (DEGs) were identified with DESeq2 (Love et al., 2014), applying thresholds of absolute log_2_ fold change ≥1 and Benjamini–Hochberg adjusted p ≤ 0.05 (Wald test).

### 2.6 Gene disease association analysis

Known PE-associated genes were retrieved from DisGeNET (v25.1.1) (Piñero et al., 2019), OMIM (Amberger et al., 2015), and ClinVar (Landrum et al., 2014). Only genes with a GDA score ≥0.7 and an evidence index ≥0.7 were included. Candidate genes were further cross-checked in GeneCards and PubMed for reported PE associations.

### 2.7 Functional profiling of DEGs

KEGG pathway and GO term enrichment (BP, MF, CC) analyses were performed using DAVID v6.8 (Huang et al., 2009). Significance was assessed by Fisher’s exact test (Fisher, 1922) with FDR-adjusted p ≤ 0.05. Results were ranked by p-value significance.

### 2.8 Protein Protein interaction (PPI) network analysis

PPI networks were generated using multiple curated databases, including STRING (Szklarczyk et al., 2023) and InnateDB (Breuer et al., 2013). InnateDB integrates interaction data from several established resources, such as IntAct (Orchard et al., 2014), MINT (Licata et al., 2012), DIP (Salwinski et al., 2004), BIND (Isserlin et al., 2011), and BioGRID (Chatr-aryamontri et al., 2015). A zero-order network (direct interactions only) was selected to reduce complexity. Hub genes were defined as nodes with ≥10 edges. Modules were identified with the WalkTrap algorithm, and significance was evaluated via the Wilcoxon rank-sum test (Haynes et al., 2013).

### 2.9 Transcriptional regulatory network analysis

Upstream transcription factors (TFs) for DEGs were identified using Expression2Kinases (X2K) (Chen et al., 2012) with the ChEA database. The Genes2Networks module linked transcription factors to PPI partners, generating transcriptional complexes. The top 10 TFs were ranked by combined p- and z-scores.

## 3 Results

### 3.1 Differential gene expression across experimental groups

**FIGURE 1:**
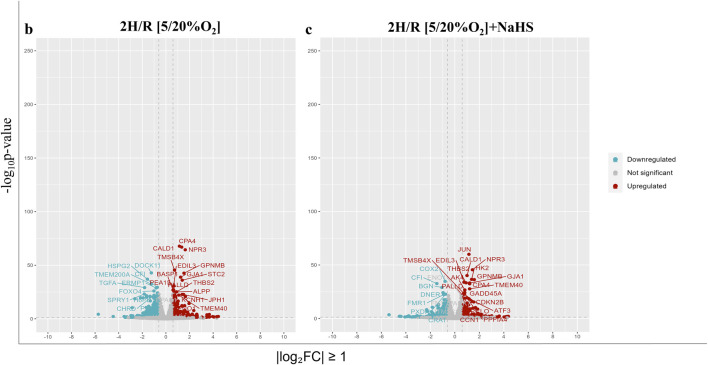
Differential gene expression in HTR-8/SVneo cells following two cycles of hypoxia/reoxygenation injury (H/R; 5/20% O_2_) with or without NaHS supplementation. Volcano plots illustrate the distribution of DEGs in group b: cells subjected to two cycles of H/R injury at 5/20%, and group c: cells supplemented with NaHS post H/R injury. Each point represents an individual gene. The x-axis shows the log_2_ fold change, while the y-axis shows −log_10_ (adjusted p-value), indicating statistical significance. Volcano plots were generated in R using the myVolcano plot function. Genes with adjusted p-value <0.05 and |log_2_ FC| ≥ 1 were considered significantly differentially expressed.

**FIGURE 2:**
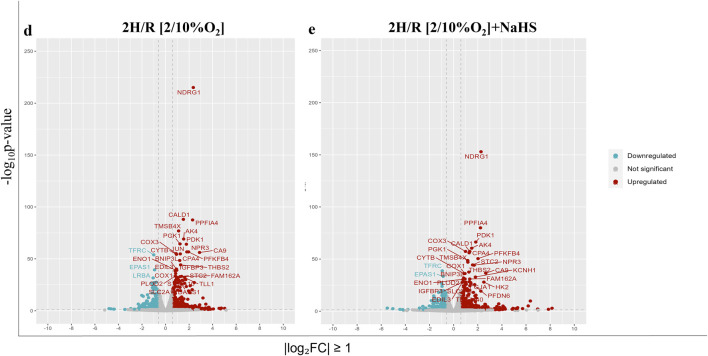
Gene expression changes in HTR-8/SVneo cells following two cycles of hypoxia/reoxygenation injury (H/R; 2/10% O_2_) with or without NaHS supplementation. Volcano plots illustrate the distribution of DEGs in group d: cells exposed to two cycles of H/R injury at 2/10% O_2_, group e: cells supplemented with NaHS post 2H/R injury. Each dot represents an individual gene. The x-axis indicates the log_2_ fold change, while the y-axis shows −log_10_ (adjusted p-value), indicating statistical significance. Volcano plots were generated in R using the myVolcano plot function. Genes with an adjusted p-value < 0.05 and |log_2_ FC| ≥ 1 were considered significantly differentially expressed.

**FIGURE 3:**
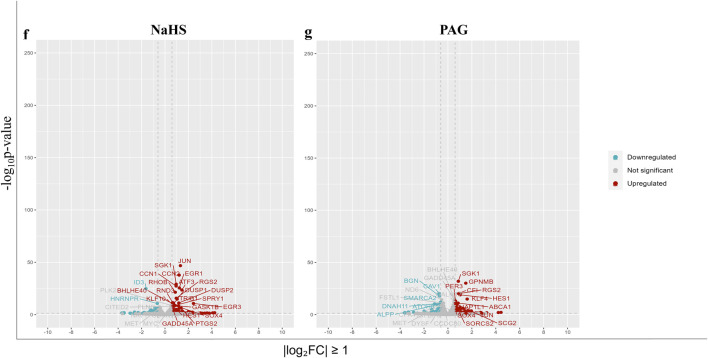
Gene expression changes following treatment with NaHS (exogenous H2S donor) and PAG (CSE inhibitor) in HTR-8/SVneo cells. Volcano plots illustrate DEGs in group f: cells supplemented with NaHS and group g: cells treated with PAG. Each dot represents an individual gene. The x-axis indicates the log_2_ fold change, while the y-axis shows −log_10_ (adjusted p-value), indicating statistical significance. Volcano plots were generated in R using the myVolcano plot function. Genes with an adjusted p-value < 0.05 and |log_2_ FC| ≥ 1 were considered significantly differentially expressed.

RNA sequencing revealed distinct and overlapping transcriptional responses to H/R stress and H_2_S modulation across experimental groups. Volcano plots (Figures 1–3) demonstrated marked transcript upregulation in H/R [5/20% O_2_] (group b) and H/R [2/10%O_2_] (group d) stress, as well as in corresponding NaHS-treated rescue groups (groups c and e).

Certain genes were consistently elevated across H/R and H_2_S treatment conditions. THBS2 was recurrently upregulated in groups b–e, suggesting a conserved role in extracellular matrix remodelling during hypoxia. Glycolysis and HIF pathway-associated genes (NDRG1, PGK1, ENO1) were strongly induced in H/R [2/10% O_2_] (groups d and e), indicating robust metabolic adaptation. CPA4 expression was broadly increased across groups b–e, while GPNMB was enriched in Groups b, c, and g, linking it to both hypoxic and endogenous H_2_S-inhibited states.

Uniform induction of the stress-responsive transcription factor, JUN, was observed in Groups c, d, f, and g, highlighting its role in H_2_S-associated signalling irrespective of oxygen levels. NaHS treatment alone (Group f) yielded a unique signature with high PTGS2, DUSP1, and GADD45A expression, exhibiting an inflammatory/stress phenotype. Groups f and g showed upregulation of SGK1, implying H_2_S-linked regulation of cellular survival pathways.

### 3.2 Overlap of differentially expressed genes

**FIGURE 4:**
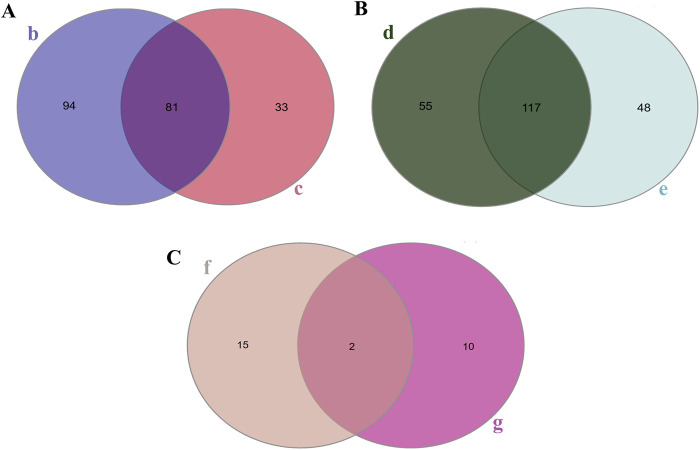
Venn diagrams illustrating the numbers of common and unique DEGs between **(A)**: Group b- 2H/R [5/20% O_2_] and Group c- 2H/R [5/20% O_2_] + NaHS; **(B)**: Group d- 2H/R [2/10% O_2_] and Group e- 2H/R [2/10% O_2_] + NaHS; and **(C)**: Group f- NaHS and Group g- PAG. DEGs were identified using thresholds of adjusted p-value ≤ 0.05 and |log_2_ Fold Change| ≥ 1. The diagrams were generated using InteractiVenn to visualize gene overlap among the experimental groups.

**FIGURE 5:**
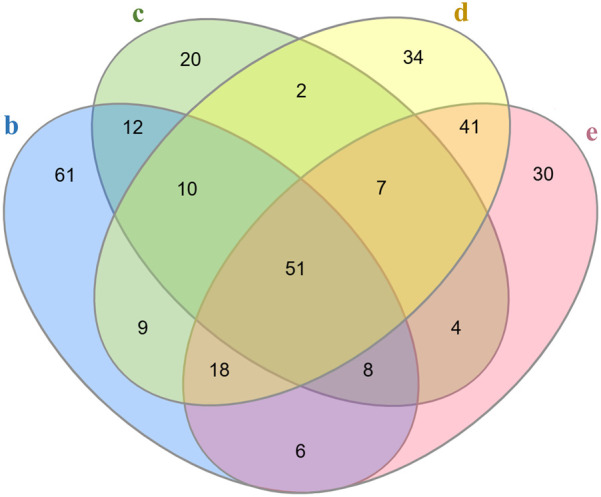
Overlap of DEGs among H/R and NaHS supplementation post H/R treatment in HTR-8/SVneo cells. The Venn diagram visualizes shared and unique DEGs across four experimental groups (b, c, d, and e). DEGs were selected using an adjusted p-value ≤ 0.05 and |log_2_ Fold Change| ≥ 1. The diagram was generated using InteractiVenn. This analysis reveals both common and condition-specific transcriptional responses to varying degrees of hypoxic injury and hydrogen sulfide-based redox modulation via NaHS. b: 2H/R [5/20% O_2_]; c: 2H/R [5/20% O_2_] + NaHS; d: 2H/R [2/10% O_2_]; e: 2H/R [2/10% O_2_] + NaHS.

Venn diagram comparisons (Figure 4) showed that in H/R [5/20%], 81 DEGs were common between injury (group b) and NaHS rescue (group c), with 94 and 33 genes unique to each group, respectively. In H/R [2/10%], 117 DEGs were shared between injury (group d) and NaHS rescue (group e) conditions, with 55 and 48 unique to each. Across groups b–e, 51 DEGs overlapped, forming a conserved hypoxia-response signature (Figure 5). NaHS-only (group f) and PAG (group g) showed minimal overlap (two genes), underscoring distinct transcriptional effects of exogenous H_2_S (NaHS supplementation) *versus* endogenous H_2_S inhibitor, which modulate largely non-overlapping gene networks, consistent with their biologically opposite roles (Figure 4).

### 3.3 Overview of transcriptional responses to H/R and H_2_S

Transcriptomic profiling across six experimental conditions revealed both oxygen-dependent and H_2_S-specific remodelling of trophoblast signalling. Both H/R models (5/20% and 2/10% O_2_) activated conserved stress-response networks, whereas NaHS and PAG produced distinct modulatory effects. Analysis of top DEGs showed prominent alterations in oxidative stress mediators (NCF2, CYBB), hypoxia-induced regulators (CA9), inflammatory transcripts (PTGS2, IL1B), ECM modulators (VCAN, THBS2), and metabolic factors (HK2, PDK1). The H/R [2/10% O_2_] + NaHS (group e) rescue produced the strongest transcriptional activation (Table 1).

**TABLE 1:** Overview of transcriptional responses across experimental conditions.

Experimental condition	Global transcriptomic pattern
Group b: H/R (5/20% O_2_)	Activation of conserved stress-response networks involving oxidative stress, metabolism, ECM remodelling, and vascular regulators
Group c: H/R (5/20%) + NaHS	Partial reprogramming of H/R-induced genes with preservation of metabolic and growth-factor signalling
Group d: H/R (2/10% O_2_)	Strong hypoxia-adaptive and oxidative transcriptomic signature with pronounced metabolic and redox remodelling
Group e: H/R (2/10%) + NaHS	Broadest transcriptional activation across oxidative, inflammatory, angiogenic, and ECM-remodelling programs
Group f: NaHS alone	Restricted induction of early stress-response and signalling genes without broad metabolic activation
Group g: PAG (CSE inhibitor)	Distinct inflammatory–angiogenic transcriptional changes with limited overlap with H/R-driven responses

### 3.4 Top five upregulated and downregulated genes across conditions

**FIGURE 6:**
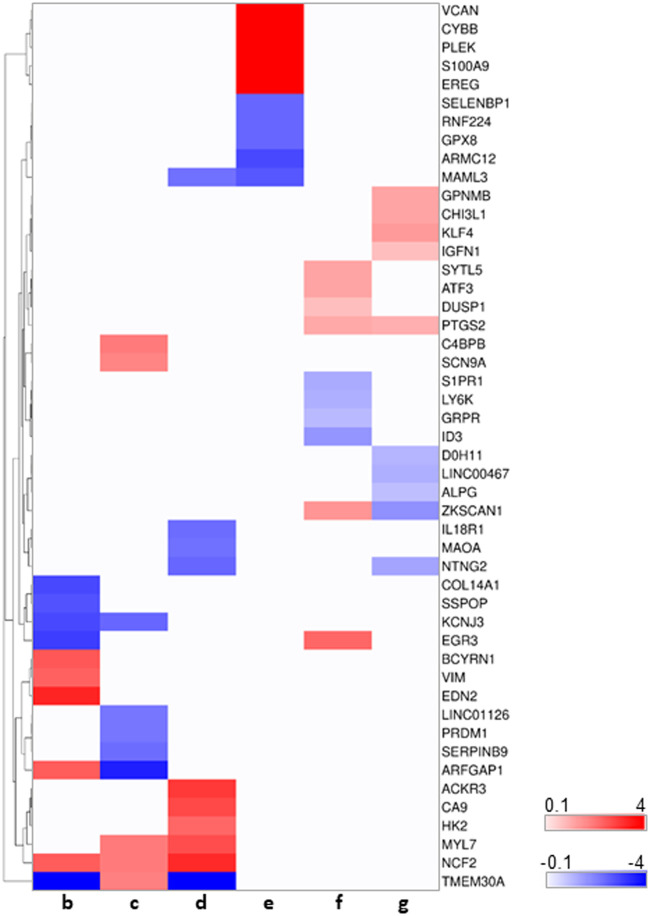
Heatmap illustrating the expression patterns of the top five most significantly upregulated and downregulated genes identified in each treatment condition (groups b to g) relative to the control. Gene expression values are shown as Z-score normalized log_2_-transformed counts, with red indicating upregulation and blue indicating downregulation. Hierarchical clustering was applied to gene expression data to visualize similarities in expression profiles relative to the control. b: 2H/R [5/20% O_2_]; c: 2H/R [5/20% O_2_] + NaHS; d: 2H/R [2/10% O_2_]; e: 2H/R [2/10% O_2_] + NaHS; f: NaHS; g: PAG.

H/R [5/20% O_2_] (group b) induced vasoregulatory and oxidative genes (EDN2, NCF2, ARFGAP1, VIM) while suppressing transcriptional and ECM components (EGR3, TMEM30A, COL14A1) (Figure 6; Table 2).

**TABLE 2:** Top five upregulated and downregulated genes across conditions.

Experimental condition	Top upregulated genes	Top downregulated genes
Group b: H/R (5/20% O_2_)	EDN2, BCYRN, NCF2, ARFGAP1, VIM	TMEM30A, EGR3, KCNJ3, COL14A1, SSPOP
Group c: H/R (5/20%) + NaHS	C4BPB, NCF2, MYL7, TMEM30A, SCN9A	ARFGAP1, KCNJ3, PRDM1 SERPINB9, LINCO1126
Group d: H/R (2/10% O_2_)	NCF2, ACKR3, CA9, MYL7, HK2	TMEM30A, NTNG2, IL18R1, MAML3, MAOA
Group e: H/R (2/10%) + NaHS	S100A9, EREG, VCAN, CYBB, PLEK	ARMC12, MAML3, GPX8, SELENBP1, RNF224
Group f: NaHS alone	EGR3, ZKSCAN1, SYLTL5, ATF3, PTGS2	ID3, S1PR1, LY6K, GRPR
Group g: PAG (CSE inhibitor)	KLF4, GPNMB, CHI3L1, PTGS2, IGFN1	ALPG, DNAH11, LINC00467, NTNG2, ZKSCAN1

NaHS post H/R [5/20% O_2_] (group c) partially reversed some H/R-driven changes. Itupregulated C4BPB, NCF2, MYL7 while downregulating ARFGAP1 and PRDM1 (Figure 6; Table 2).

H/R [2/10%O_2_] (group d) produced a strong oxidative and hypoxia-response signature (NCF2, ACKR3, CA9, HK2) and repressed IL18R1, MAML3, NTNG2 (Figure 6; Table 2).

NaHS after H/R [2/10% O_2_] (group e) showed the broadest transcriptional induction including S100A9, EREG, VCAN, CYBB, PLEK and suppression of mitochondrial/Notch related regulators (MAML3, GPX8) (Figure 6; Table 2).

NaHS alone (group f) upregulated immediate-early/stress response genes (PTGS2, ATF3, EGR3) while downregulating ID3 and S1PR1. PAG treatment (group g) induced KLF4, CHI3L1, PTGS2, and reduced NTNG2, DNAH11, ZKSCAN1.

### 3.5 Pathway-level reprogramming

**FIGURE 7:**
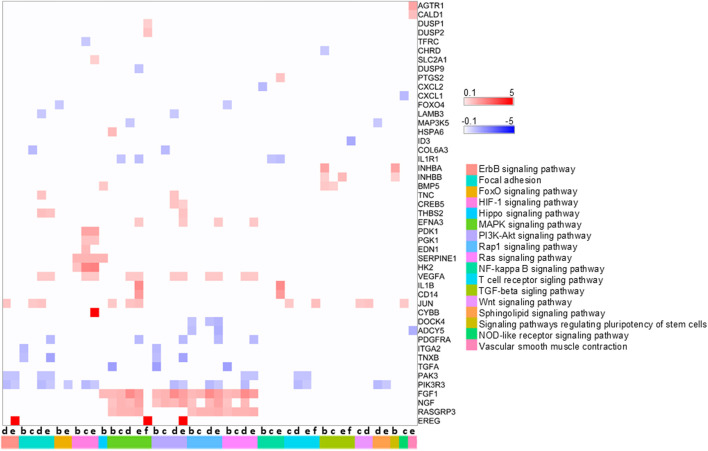
Heatmap highlighting pathways enriched across treatment groups driven by H/R injury and exogenous treatments (NaHS and PAG). Each column denotes an enriched pathway within a treatment group, and each row represents an individual gene. Color intensity represents differential gene expression across experimental groups, with red indicating upregulation and blue indicating downregulation. b: 2H/R [5/20% O_2_]; c: 2H/R [5/20% O_2_] + NaHS; d: 2H/R [2/10% O_2_]; e: 2H/R [2/10% O_2_] + NaHS; f: NaHS; g: PAG.

Across conditions, DEGs were enriched in multiple signalling pathways including ErbB, PI3K–Akt, MAPK, HIF-1, FoxO, Rap1/Ras, NF-κB, Hippo, TGF-β, Wnt, focal adhesion, NOD-like receptor, sphingolipid signalling, and pluripotency pathways. In H/R [5/20% O_2_] (group b) enrichment of Rap1, PI3K–Akt, MAPK, FoxO, and NF-κB pathways indicated coordinated activation of pro-survival, angiogenic, and cytoskeletal remodelling responses. NaHS treatment post H/R [5/20% O_2_] [group c) preserved core pathways but appear to shift signalling balance, maintaining metabolic and growth-factor cascades while modulating inflammatory responses. H/R [2/10% O_2_] (group d) showed strong enrichment of PI3K–Akt, MAPK, Rap1/Ras, focal adhesion, and sphingolipid pathways consistent with enhanced metabolic, adhesive, and redox adaptation. H/R [2/10% O_2_] + NaHS (group e) maintained the H/R-induced signalling backbone while strengthening NF-κB and TGF-β, suggesting expanded remodelling capacity under severe stress. NaHS alone (group f) showed enrichment primarily in MAPK, TGF-β, and T-cell receptor pathways, reflecting early stress-kinase activation without broad metabolic rewiring. In contrast, PAG (group g) showed minimal enrichment of trophoblast relevant pathways, suggesting weaker global signalling alterations (Figure 7) (Table 3).

**TABLE 3:** Pathway-level reprogramming across experimental groups.

Experimental condition	Major enriched pathways
Group b: H/R (5/20% O_2_)	Rap1, ErbB, PI3K–Akt, MAPK, FoxO, NF-κB
Group c: H/R (5/20%) + NaHS	PI3K–Akt, MAPK, focal adhesion, HIF-1, NF-kb, T cell receptor, TGF-b, Wnt
Group d: H/R (2/10% O_2_)	ErbB, PI3K–Akt, MAPK, Rap1/Ras, focal adhesion, sphingolipid signalling, T cell receptor, Wnt
Group e: H/R (2/10%) + NaHS	HIF-1, NF-κB, TGF-β, Hippo, ErbB, focal adhesion, FoxO, MAPK, PI3K-Akt, Ras, T cell receptor
Group f: NaHS alone	MAPK, TGF-β, T-cell receptor signalling
Group g: PAG (CSE inhibitor)	No dominant enrichment among shortlisted trophoblast pathways

### 3.6 Biological processes

**FIGURE 8:**
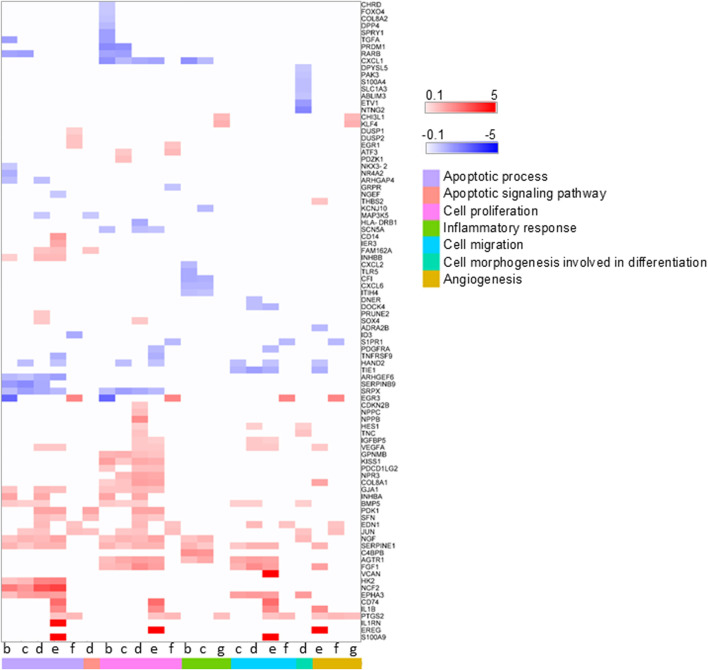
Heatmap of the biological processes (BP) highlighting key gene expression changes across experimental groups. Each column denotes an enriched BP term within a treatment group, and each row represents an individual gene. Color intensity represents differential gene expression across experimental groups, with red indicating upregulation and blue indicating downregulation. b: 2H/R [5/20% O_2_]; c: 2H/R [5/20% O_2_] + NaHS; d: 2H/R [2/10% O_2_]; e: 2H/R [2/10% O_2_] + NaHS; f: NaHS; g: PAG.

Biological process analysis revealed coordinated transcriptional programs related to cell growth, survival, migration, differentiation, angiogenesis, and inflammation. Under both H/R conditions, apoptotic signalling showed a consistent upregulation of pro-survival and metabolic genes, such as HK2, NGF, SERPINE1, VEGFA and PDK1, while suppressing multiple pro-apoptotic mediators, including HAND2 (group c), MAP3K5 (group d), and TNFRSF9 (group e). The strongest anti-apoptotic signature was observed in H/R [2/10% O_2_] with or without NaHS. In contrast, NaHS alone primarily induced the stress-responsive genes without apparent apoptotic engagement. Cell-proliferation processes were characterised by upregulation of growth and survival regulators (FGF1, NGF, BMP5, and IGFBP5) and downregulation of inhibitory factors such as PRDM1, SPRY1, FOXO4, with the broadest proliferative profile evident under H/R [2/10% O_2_]; NaHS treatment further enhanced the expression of immune-associated proliferative genes such as EREG, PTGS2, IL1B, and PDCD1LG2. Inflammatory-response genes displayed a context-dependent pattern, with H/R [5/20% O_2_] (group b) suppressing acute chemokines like CXCL1/2/6, TLR5. PAG treatment promoted a chronic inflammatory–remodelling signature centred on PTGS2, KLF4, and CHI3L1. M igration related processes involved induction of ECM and motility-associated genes like EPHA3, SERPINE1 and BMP5, with strong VEGFA/IGFBP5 induction under H/R [2/10% O_2_] (group d), concurrent with downregulation of endothelial and guidance cues such as TIE1, PDGFRA, DOCK4 andS1PR1. NaHS treatment further expanded this migratory transcriptomic repertoire. Morphogenesis-related differentiation pathways were selectively modulated under H/R [2/10% O_2_] (group d) with induction of EPHA3, NGF, JUN and BMP5 together with suppression of neuronal or axon-guidance genes like NTNG2 and ETV1. Angiogenesis-related processes were strongly enriched in H/R [2/10% O_2_] with NaHS (group e), involving induction of EREG, PTGS2, VEGFA and THBS2 coupled with downregulation of stabilising or vasoconstrictive genes like TIE1, HAND2, ADRA2B. PAG treatment again elicited an inflammatory angiogenic profile driven by PTGS2, KLF4, and CHI3L1 (Figure 8) (Supplementary Table S1).

### 3.7 Integrated interpretation

Collectively, the DEG, pathway, and biological process analyses suggest that H/R is associated with a transcriptional shift towards a hypoxia-adapted and plastic trophoblast phenotype. This includes metabolic reprogramming (HIF-1 axis), ECM, growth factor signalling, reduced apoptosis, and enhanced angiogenic and migratory capacity.

H_2_S appears to act as a context-dependent modulator of these responses. It influences inflammatory signalling, growth factor pathways, and metabolic adaptation, with more pronounced effects under severe hypoxic stress.

### 3.8 JUN and PTGS2 emerged as a candidate transcriptomic nodes in trophoblast stress and redox signalling

**FIGURE 9:**
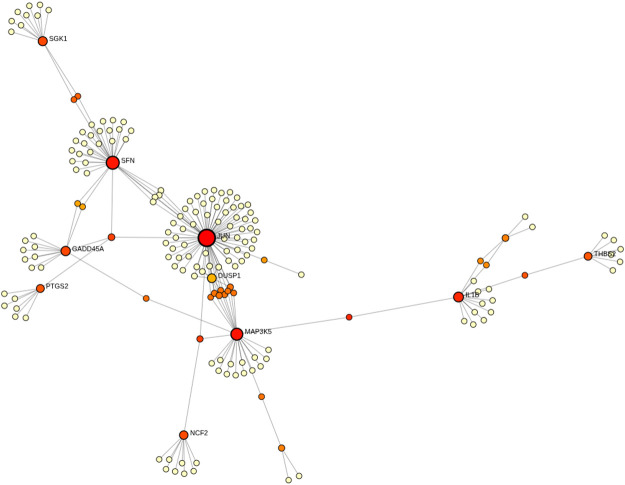
Protein–protein interaction (PPI) network showing hub genes identified from RNA-seq data constructed using the STRING database and visualized in Cytoscape. Nodes represent proteins encoded by DEGs, while edges denote predicted or known interactions. The hub genes, including JUN, MAP3K5, DUSP1, SFN, IL1B, THBS2, GADD45A, NCF2, SGK1, and PTGS2, were identified using network topology analysis with the degree centrality algorithm. Node color intensity correlates with connectivity degree, with red nodes indicating the highest degree (most interactions).

To identify central regulatory nodes governing trophoblast responses to hypoxia and H_2_S modulation, a PPI network was constructed using STRING and visualized in Cytoscape (Figure 9). Topological analysis (degree centrality ≥10) revealed ten hub genes: JUN, MAP3K5, DUSP1, SFN, IL1B, THBS2, GADD45A, NCF2, SGK1, and PTGS2. These nodes formed interconnected modules associated with oxidative stress, inflammatory signalling, apoptosis, and ECM remodelling. JUN emerged as the most highly connected transcriptional regulator and upregulated under both NaHS treatment (group f) and H/R [2/10% O_2_] (group d). JUN was enriched across multiple signalling pathways, including MAPK, ErbB, Wnt, NOD-like receptor, focal adhesion, and T cell receptor pathways, indicating a central role in coordinating redox adaptation, transcriptional reprogramming, cell survival, migration, and early inflammatory signalling. PTGS2 was also a major hub and was upregulated in NaHS-treated and PAG-treated conditions (Figure 6), suggesting that both exogenous and endogenous H_2_S influence prostaglandin-mediated stress and inflammatory responses. PTGS2 displayed significant enrichment within the NF-κB pathway, syncing with its functional engagement in cytokine-driven vascular malfunction, angiogenic, and apoptosis modulation. Other hub genes contributed to stress kinase signalling (**MAP3K5, GADD45A, DUSP1**), cytoskeletal regulation (**SFN, SGK1**), and oxidative responses (**NCF2**). **THBS2** linked ECM remodelling with angiogenic processes. Altogether, the PPI analyses showed that trophoblast response to H/R and H_2_S is directed by a precise, highly interconnected set of stress-responsive transcriptional and signalling hub genes. Based on transcriptomic and network analyses, JUN and PTGS2 are identified as candidate hub genes; however, they should be interpreted as computationally predicted regulators whose functional roles remain to be experimentally validated.

## 4 Discussion

In this study, RNA-seq was employed to explore the influence of H_2_S on trophoblast transcriptional responses to H/R, a key driver of placental oxidative stress. Two relevant oxygen-fluctuation paradigms were combined with an exogenous H_2_S donor (NaHS) and pharmacologic CSE inhibition (PAG). These datasets were then examined using differential expression, pathway enrichment, biological processes, and PPI network analysis. Collectively, our data suggests that H/R fine-tunes trophoblasts toward a hypoxia-adapted, invasive, angiogenic phenotype. Importantly, given that these observations are derived from transcriptomic profiling in an *in vitro* model, they should be interpreted as associative and hypothesis-generating, rather than indicative of direct mechanistic causality.

H_2_S appears to act as a context-dependent modulator, and influencing metabolic, inflammatory, apoptotic, and vascular pathways rather than exerting a uniformly cytoprotective effect. JUN and PTGS2 emerged as candidate transcriptional hubs linking redox signalling with inflammation and vascular remodelling.

### 4.1 H/R paradigms recapitulate key features of placental hypoxic stress

The 2 H/R conditions were designed to model dynamic changes in oxygen availability during placental development, rather than a static hypoxic state. In early pregnancy, trophoblasts are exposed to low oxygen levels (∼2–3% O_2_), which increase to ∼8–10% O_2_ with the establishment of maternal circulation. Accordingly, the 2/10% O_2_ paradigm reflects a physiological transition, enabling assessment of adaptive trophoblast responses to changing oxygen tension. In contrast, the 5/20% O_2_ paradigm represents a relative hyperoxic shift, as oxygen levels around ∼5% O_2_ approximate physiological placental conditions, whereas 20% O_2_ corresponds to ambient atmospheric levels commonly used in standard cell culture and is relatively hyperoxic compared to the *in vivo* environment. Given that trophoblasts are highly sensitive to both oxygen levels and fluctuations, these models allow evaluation of how varying oxygen dynamics influence trophoblast behaviour through HIF-dependent and redox-mediated pathways (Burton et al., 2021). This design aligns with previous *in vivo* and *in vitro* findings showing that sustained or intermittent hypoxia modulates trophoblast differentiation, invasion, and endocrine profiles through HIF-dependent pathways and oxidative stress (Highet et al., 2015). As expected, both H/R paradigms activated a classic hypoxia stress signature, including HIF-1-linked glycolytic (HK2, PGK1, ENO1, CA9), ROS-associated regulators (NCF2), and ECM remodellers such as THBS2 and VCAN.

H/R [2/10% O_2_] (group d) produced the most pronounced metabolic and redox adaptation, consistent with studies showing that deeper hypoxia drives stronger HIF activation and metabolic reprogramming in trophoblasts (Knyazev et al., 2019).

Both the H/R conditions favoured the biological process of survival over cell death, with coordinated upregulation of pro-survival and metabolic genes (HK2, PDK1, INHBA/B, VEGFA, FAM162A) and downregulation of several pro-apoptotic mediators (MAP3K5, TNFRSF9, SRPX, SERPINB9). This predominance of the survival profile is consistent with the concept that trophoblasts initially attempt to adapt to intermittent H/R cycles, characteristic of early placental malperfusion by enhancing glycolysis, antioxidant defences, and tissue remodelling, and undergo apoptosis only when these compensatory mechanisms fail (Aouache et al., 2018). It is important to note that these interpretations are derived from an *in vitro* trophoblast model and transcriptomic data alone. The absence of protein-level validation, direct measurements of H_2_S or oxidative stress, and functional assays limits the ability to establish causality or define precise mechanistic pathways. These findings should therefore be interpreted within the context of an exploratory systems-level analysis.

### 4.2 H_2_S as a context-dependent modulator of trophoblast stress responses

These observations support and extend our previous work implicating dysregulated H_2_S signalling in PE and other placental disorders. Studies, including those from our group, have reported reduced levels of placental and circulating H_2_S in women with PE, accompanied by decreased CSE and/or CBS expression in placental tissues, while CSE-deficient models exhibit impaired placental vascularisation and adverse pregnancy outcomes (Wang et al., 2013; Gupta et al., 2023; Rezai et al., 2021). However, genome-wide trophoblast responses to H/R in the context of H_2_S modulation have remain poorly defined. In our model, NaHS post H/R (Groups c and e) did not simply reverse injury but reprogrammed several pathways. After H/R [5/20% O_2_] treatment with NaHS largely preserved the anti-inflammatory and repair-oriented state with sustained downregulation of chemokines and complement components, alongside maintenance of genes such as AGTR1, NGF, C4BPB, and SERPINE1.

Post H/R [2/10% O_2_] treatment, NaHS strengthened HIF-1–linked glycolysis, angiogenic signalling (VEGFA, EREG, COL8A1), and ECM remodelling (VCAN, THBS2), while still maintaining an anti-apoptotic profile. At the same time, NaHS permitted a late inflammatory amplification (IL1B, PTGS2, CD74), suggesting that H_2_S favours an adaptive, angiogenic inflammatory programme rather than blanket suppression of inflammation in this context. By contrast, PAG-treated cells (group g) showed a distinct, more chronic inflammatory angiogenic signature centred on PTGS2, KLF4 and CHI3L1, without the broad hypoxia-adaptation seen with H/R.

This aligns with the idea that endogenous CSE-derived H_2_S exerts anti-inflammatory effects in pregnancy and that its loss contributes to low-grade vascular inflammation and maladaptive remodelling, hallmarks of PE (Du et al., 2025). Together, donor and inhibitor data emphasise that H_2_S emerges as a context-dependent and potentially biphasic modulator, exerting differential effects depending on the severity of hypoxic stress and the underlying cellular state, rather than functioning as a uniformly protective factor. These findings suggest that H_2_S-mediated signalling cannot be interpreted as universally cytoprotective but instead reflects a nuanced balance between adaptive metabolic and angiogenic responses and context-specific inflammatory activation. This interpretation is in line with previous work by Hua Wang et al., (Hua et al., 2013), which demonstrates that H_2_S exerts a biphasic regulatory role in inflammation, characterized by pro-inflammatory effects in acute, neutrophil-driven responses and anti-inflammatory effects during later, lymphocyte-associated phases.

Notably, no direct biochemical measurements of H_2_S levels or redox status were performed in this study, which limits mechanistic interpretation of these effects.

### 4.3 Coordinated regulation of migration, morphogenesis, and angiogenesis

Together, the DEG, pathway, and GO results suggest that H/R ± NaHS drive a coordinated shift toward an invasive, vessel-remodelling trophoblast phenotype. Under H/R [2/10% O_2_], genes promoting migration and morphogenesis (EPHA3, FGF1, AGTR1, BMP5, HES1, TNC) were induced, while neuronal/axon-guidance and metastasis-type genes (NTNG2, ETV1, S100A4, PAK3) were repressed, indicating a refined, trophoblast-appropriate morphogenetic programme. This aligns with existing work showing that oxygen tension and oxidative stress dynamically regulate trophoblast invasion and differentiation (Mukherjee et al., 2021).

Angiogenic related processes were maximally enriched in H/R [2/10%O_2_] + NaHS (group e) with induction of VEGFA, EREG, PTGS2, IL1B, THBS2, SERPINE1, and COL8A1 alongside suppression of vessel-stabilising genes (TIE1, HAND2, ADRA2B). These changes suggest a state of active sprouting and vascular remodelling with reduced vasoconstrictive tone, precisely the balance required for spiral artery transformation. The observation that PAG also upregulated PTGS2 and CHI3L1, outside of a robust hypoxia-adapted background, is consistent with reports linking COX-2 overexpression and inflammatory vascular changes to adverse pregnancy outcomes and PE-like features in animal and human studies (Sones et al., 2016).

### 4.4 JUN and PTGS2 as convergent hubs of trophoblast stress and redox signalling

One of the strengths of this study is the convergence of JUN and PTGS2 across multiple analytic layers. Both genes were (i) significantly upregulated in several treatment groups, (ii) enriched in numerous stress and inflammatory pathways, (iii) associated with key biological processes (apoptosis, proliferation, morphogenesis, angiogenesis, inflammatory response), and (iv) identified as high-degree hubs in the PPI network.

AP-1 family members, including JUN, are known regulators of trophoblast differentiation and invasion, with dynamic changes in AP-1 composition influencing endocrine vs. invasive lineages and responses to hypoxia (Kubota et al., 2015; Yuen et al., 2013). Our data extend this by showing that JUN sits at the centre of an integrated stress-kinase module (MAP3K5, GADD45A, DUSP1, SFN) and is recruited by both NaHS and H/R [2/10%O_2_], highlighting its role as a redox-responsive transcriptional hub in H_2_S-modulated trophoblast adaptation.

PTGS2 (COX-2) has a complex relationship with placental pathology: it is differentially expressed in PE placentae, and both excessive and insufficient COX-2 activity have been implicated in vascular dysfunction and PE (Börekçi et al., 2006; Goksu Erol et al., 2012; Khan et al., 1999). Our finding that PTGS2 is induced by NaHS and by CSE inhibition but in distinct network contexts (adaptive angiogenic vs. chronic inflammatory) suggests that PTGS2 may integrate H_2_S-dependent signals with broader inflammatory and vascular responses. These observations resonate with recent bioinformatic studies that also identify PTGS2 as a hub in PE-associated placental networks (Azmi et al., 2024).

Taken together, JUN and PTGS2 appear to function as key integrators of trophoblast responses to fluctuating oxygen and H_2_S availability. While the present findings are transcriptomic and hypothesis-generating, they highlight AP-1 and COX-2 signalling, along with their upstream regulators, as could be a promising avenue for mitigating oxidative and inflammatory damage in placental ischemia. Despite these important findings, several limitations of the present study should be acknowledged. First, the work is based on a single trophoblast cell line and an acute *in vitro* H/R model, which cannot fully recapitulate the cellular diversity, maternal-fetal cross-talk, and chronic nature of *in vivo* placental ischemia. While such models provide a controlled and reproducible framework for mechanistic exploration, *in vivo* validation remains essential. Second, we did not directly measure H_2_S levels, CSE/CBS activity, or oxidative stress markers; therefore, the link between transcriptomic changes and underlying biochemical fluxes remains inferential. Third, although RNA sequencing enables comprehensive assessment of steady-state mRNA abundance, we did not validate protein expression, post-translational modifications (e.g., JUN phosphorylation, COX-2 activity), or perform functional assays of trophoblast invasion, proliferation, or angiogenesis. Finally, bulk RNA-seq does not resolve potential cellular heterogeneity within the HTR-8/SVneo population that may differentially respond to H/R and H_2_S modulation. Accordingly, these findings should be interpreted as hypothesis-generating and warrant further validation using protein-level, functional, and higher-resolution approaches, including studies in trophoblast systems and clinical samples.

Future studies integrating functional, single-cell approaches will be critical to resolve cell-type–specific responses and validate the mechanistic role of H_2_S signaling in placental pathology.

In conclusion, this study provides a transcriptome-wide characterization of how H_2_S modulates gene networks in H/R stressed trophoblasts, highlighting key roles for JUN and PTGS2-centered pathways in redox regulation, inflammation, and angiogenic balance. These findings support a potential role for H_2_S in shaping trophoblast adaptation to placental ischemic stress and offer mechanistic insight into its contribution to the pathophysiology of PE. However, the context-dependent induction of inflammatory pathways and PTGS2 underscores the need for caution, as indiscriminate targeting of H_2_S signaling may produce unintended effects. Further studies are required to define the optimal conditions, dosage, and tissue-specific responses before translating these findings into therapeutic strategies.

## Data Availability

The datasets presented in this study can be found in SRA database repository under Accession number: PRJNA1395812.
