# Supplementary material for: Hydrogen sulfide modulates gene networks in hypoxia/reoxygenation-stressed trophoblasts: insights from transcriptome profiling
Source: Front Bioinform. 2026 Jun 16;6:1785302. doi: 10.3389/fbinf.2026.1785302 (PMC13314452; doi:10.3389/fbinf.2026.1785302)
Supplement: Supplementary file 1 [file Supplementaryfile1.docx]

**Table S1**

**Apoptosis**

| **Condition** | **Upregulated genes** | **Downregulated genes** | **Net biological trend** |
| --- | --- | --- | --- |
| Group b: H/R (5/20%) | NCF2, HK2, EPHA3, SERPINE1, NGF, BMP5, INHBA, GJA1, INHBB | EGR3, SRPX, SERPINB9, ARHGEF6, NR4A2, NKX3-2, PDZK1, RARB, TGFA | Survival-biased transcription with suppression of pro-apoptotic mediators |
| Group c: H/R (5/20%) + NaHS | NCF2, HK2, EPHA3, SERPINE1, NGF, JUN, BMP5 | SRPX, SERPINB9, ARHGEF6, HAND2, RARB | Maintained anti-apoptotic shift with early stress-response activation |
| Group d: H/R (2/10%) | NCF2, HK2, EPHA3, SERPINE1, NGF, JUN, EDN1, SFN, PDK1, BMP5, INHBA, GJA1, VEGFA, INHBB, FAM162A, SOX4, PRUNE2 | SRPX, SERPINB9, ARHGEF6, MAP3K5, NR4A2 | Strongest suppression of apoptotic signaling |
| Group e: H/R (2/10%) + NaHS | S100A9, IL1RN, PTGS2, IL1B, CD74, EPHA3, NCF2, HK2, SERPINE1, NGF, SFN, VEGFA, PDK1, GJA1, INHBB, FAM162A, IER3 | SRPX, ARHGEF6, HAND2, TNFRSF9, NGEF | Anti-apoptotic profile with late inflammatory activation |
| Group f: NaHS | PTGS2, JUN, EDN1, EGR3, DUSP1, DUSP2, EGR1 | ID3 | Stress-response activation without apoptotic commitment |

**Apoptotic signalling pathway**

| **Condition** | **Upregulated genes** | **Downregulated genes** | **Net biological trend** |
| --- | --- | --- | --- |
| Group d: H/R (2/10%) | JUN, SFN, PDK1, FAM162A | MAP3K5 | Suppression of stress-kinase–driven apoptosis |

**Cell proliferation**

| **Condition** | **Upregulated genes** | **Downregulated genes** | **Net biological trend** |
| --- | --- | --- | --- |
| Group b: H/R (5/20%) | FGF1, AGTR1, SERPINE1, NGF, BMP5, INHBA, GJA1, COL8A2 | EGR3, SRPX, SCN5A, CXCL1, RARB, PRDM1, TGFA, FOXO4, CHRD, SPRY1, DPP4, COL8A2 | Growth-permissive with inhibition of differentiation brakes |
| Group c: H/R (5/20%) + NaHS | FGF1, SERPINE1, NGF, JUN, NPR3, KISS1, GPNMB, AGTR1, BMP5, GJA1, COL8A2, PDZK1, ATF3 | SRPX, HAND2, CXCL1, RARB, PRDM1 | Sustained proliferative signaling |
| Group d: H/R (2/10%) | FGF1, AGTR1, NGF, JUN, SFN, VEGFA, TNC, IGFBP5, HES1, SERPINE1,  EDN1, PDK1, BMP5, INHBA, GJA1, COL8A2, NPR3, PDCD1LG2, KISS1, GPNMB, HES1, NPPB, NPPC, CDKN2B, SOX4 | SRPX, CXCL1, HLA-DRB1, SCN5A | Broad proliferative expansion |
| Group e: H/R (2/10%) + NaHS | EREG, PTGS2, IL1B, FGF1, SERPINE1, VEGFA, CD74, AGTR1, NGF, SFN, PDK1, GJA1, COL8A2, NPR3, PDCD1LG2, KISS1, GPNMB, VEGFA, IGFBP5 | SRPX, PDGFRA, CXCL1, HAND2, TNFRSFN9, SCN5A | Angiogenic-linked proliferation |
| Group f: NaHS | PTGS2, JUN, EGR3, ATF3, EDN1, EGR1 | S1PR1, GRPR | Restricted proliferative activation |

**Inflammatory response**

| **Condition** | **Upregulated genes** | **Downregulated genes** | **Net biological trend** |
| --- | --- | --- | --- |
| Group b: H/R (5/20%) | AGTR1, C4BPB, SERPINE1, NGF | CXCL1, CXCL2, CXCL6, ITIH4, CFI, TLR5 | Suppressed acute inflammation |
| Group c: H/R (5/20%) + NaHS | AGTR1, SERPINE1, NGF, C4BPB | CXCL1, CXCL6, CFI, KCNJ10, ITIH4 | Anti-inflammatory modulation |
| Group g: PAG | PTGS2, KLF4, CHI3L1 | — | Chronic inflammatory remodeling |

**Cell Migration**

| **Condition** | **Upregulated genes** | **Downregulated genes** | **Net biological trend** |
| --- | --- | --- | --- |
| Group c: H/R (5/20%) + NaHS | EPHA3, FGF1, SERPINE1, BMP5, AGTR1 | HAND2, TIE1 | Directed migration induction |
| Group d: H/R (2/10%) | EPHA3, FGF1, AGTR1, EDN1, VEGFA, SERPINE1, BMP5, HES1, IGFBP5 | TIE1, DOCK4, DNER | Enhanced invasive capacity |
| Group e: H/R (2/10%) + NaHS | S100A9, PTGS2, EPHA3, VCAN, VEGFA, IL1B, CD74, FGF1, AGTR1, SERPINE1, IGFBP5 | TIE1, HAND2, PDGFRA, DOCK4 | Maximal migratory remodeling |
| Group f: NaHS | PTGS2, EDN1, EGR3 | S1PR1 | Limited chemotactic activation |

**Angiogenesis**

| **Condition** | **Upregulated genes** | **Downregulated genes** | **Net biological trend** |
| --- | --- | --- | --- |
| Group e: H/R (2/10%) + NaHS | EREG, PTGS2, IL1B, VEGFA, COL8A1, THBS2, FGF1, SERPINE1 | TIE1, HAND2, ADRA2B | Strong pro-angiogenic response |
| Group f: NaHS | PTGS2, EDN1, JUN, EGR3 | S1PR1 | Partial angiogenic signaling |
| Group g: PAG | PTGS2, KLF4, CHI3L1 | — | Inflammation-associated angiogenesis |
